# Presepsin is a biomarker that can predict mortality in sepsis patients

**DOI:** 10.1590/1806-9282.20241262

**Published:** 2025-03-31

**Authors:** Naim Uzun, Adem Keskin, Recai Aci, Melek Bilgin, Sunay Akgun

**Affiliations:** 1 Agri Ibrahim Cecen University, Faculty of Pharmacy, Department of Clinical Pharmacy, Department of Pharmacy Vocational Sciences – Ağrı, Turkey.; 2 Aydin Adnan Menderes University, Institute of Health Sciences, Department of Medicine Biochemistry – Aydın, Turkey.; 3 Aydin Adnan Menderes University, Aydin Vocational School of Health Services, Department of Health Services and Techniques – Aydın, Turkey.; 4 Aydin Adnan Menderes University, Soke Vocational School of Health Services, Department of Operating Room Services – Aydın, Turkey.; 5 Samsun Training and Research Hospital, Department of Medical Microbiology – Samsun, Turkey.; 6 Samsun Training and Research Hospital, Department of Anesthesiology and Reanimation – Samsun, Turkey.

**Keywords:** Presepsin, Sepsis, Mortality, Sensitivity, Specificity, Predictive value

## Abstract

**OBJECTIVE:**

Predicting the prognosis of sepsis, a major health problem worldwide, is vital to guide the treatment process accordingly. The aim of this study was to evaluate the ability of presepsin levels to predict mortality in patients with sepsis.

**METHODS:**

The study included 87 intensive care unit patients with sepsis, 30 of whom survived. Complete blood count, blood gas, C-reactive protein, procalcitonin, albumin, and presepsin levels were analyzed. Binary logistic regression and receiver operating characteristic analyses were performed for presepsin levels.

**RESULTS:**

Presepsin levels were higher in non-survivors than in survivors. There was no significant difference in other laboratory parameters. The predictive value of presepsin level on mortality was found to be 78.20%. The cutoff value in the receiver operating characteristic curve graph for presepsin levels is 612.70 pg/mL. The positive predictive value of presepsin levels in terms of mortality is 0.5735, and the negative predictive value is 0.8512. The sensitivity of presepsin levels in terms of mortality is 73.70%, and the specificity is 73.30%. The area under the curve value in the receiver operating characteristic curve plot for mortality for presepsin levels is 0.819.

**CONCLUSION:**

Presepsin levels may predict mortality in patients with sepsis. Presepsin levels above the cutoff value of 612.70 pg/mL may be considered a risk factor for mortality in patients with sepsis.

## INTRODUCTION

Infections are a common health problem that can affect people of all ages. In general, the immune response to infections is adequate, and less treatment is needed. However, the response to infection can sometimes be inadequate and lead to organ dysfunction, a condition known as sepsis^1^. Sepsis remains the leading cause of infection-related deaths and is recognized as a global health problem^2^. As a dysregulated host response to infection, sepsis causes significant morbidity. Patients in shock due to sepsis experience circulatory, cellular, and metabolic abnormalities, leading to increased mortality^3^.

Sepsis, which remains one of the leading causes of death in intensive care unit (ICU) patients, is a heterogeneous health problem. This heterogeneity prevents the generation of reproducible figures on mortality risk. Mortality rates due to this health problem range from 15 to 56%^4^. Several screening tools are available to predict mortality and identify sepsis, such as Systemic Inflammatory Response Syndrome (SIRS) criteria and rapid Sequential Organ Failure Assessment (qSOFA) score. However, the sensitivity and specificity of these screening tools are not perfect and should be used with caution^5^. The neutrophil/lymphocyte ratio, an easily available marker in patients with sepsis, has been proposed as a useful diagnostic indicator. However, this ratio is inadequate as a diagnostic indicator, and only a combination of several biomarkers will improve diagnostic accuracy^6^. A recent meta-analysis of 60 studies involving the assessment of 99 biomarkers as independent prognostic factors for mortality in sepsis showed that isolated measurements of C-reactive protein, procalcitonin, interleukin-6, and presepsin at baseline did not help predict mortality in these patients. Furthermore, the role of these biomarkers should be evaluated in new studies measuring biomarker outcomes^7^.

CD14, which is responsible for intracellular transduction of endotoxin signals, is found on macrophages, monocytes, and granulocytes and their cell membranes. The soluble part of CD14 is found in the blood, and this soluble CD14 subtype (sCD14-ST) is known by the generic name presepsin. Presepsin is produced in association with infection and is specifically expressed in sepsis^8^. Presepsin is a promising biomarker for the triage and early diagnosis of sepsis, a life-threatening condition characterized by organ dysfunction^9^. The reference range for presepsin, the soluble part of CD14, a multifunctional glycoprotein expressed on the surface of innate immune cells, is between 60 and 382 pg/mL in healthy adults. Elevated plasma presepsin levels have been shown to be a valuable biomarker for the diagnosis of sepsis in adults. Elevated plasma presepsin levels have also been reported in patients undergoing cardiac and noncardiac surgery^10^.

Sepsis is a serious, life-threatening condition. Therefore, early diagnosis and treatment of sepsis are critical. Presepsin levels offer a promising approach to the diagnosis of sepsis and assessment of disease progression. However, presepsin has not been reported to help predict mortality in these patients. Furthermore, presepsin levels in healthy adult humans appear to be within a certain reference range. The aim of this study was to investigate the cutoff value for elevated presepsin levels and the sensitivity, specificity, and predictive values of this cutoff value for predicting mortality in patients with sepsis. Therefore, this study aims to evaluate presepsin levels as a quantitative predictor of mortality in sepsis patients.

## METHODS

### Study design

The ethics committee decision required for the study was obtained from the Non-Interventional Clinical Research Ethics Committee of Samsun University Faculty of Medicine (Protocol number: GOKAEK/2024/3/14).

Patients who were admitted to the ICUs of Samsun University Medical Faculty Training and Research Hospital with a diagnosis of sepsis between January 1, 2024, and March 31, 2024, were included in the study. Patients with comorbid cancer and patients admitted to pediatric ICUs were not included in the study. Blood samples were collected on the first day of admission to the ICU after patients/relatives were informed about the study and informed consent was obtained. Blood samples were centrifuged at 3,000 rpm, and serum samples were collected. The laboratory values analyzed on that day [white blood cell count, neutrophil count, lymphocyte count, C-reactive protein (CRP), albumin, procalcitonin, lactate (blood gas), pH, oxygen saturation, arterial partial pressure of oxygen, and base deficit] were obtained from the hospital information management system.

### Biochemical processes

Blood gas parameters were measured on an ABL90 FLEX analyzer (Radiometer Medical Supplies, Istanbul, Turkey), complete blood count parameters on a Sysmex XN-1000™ hematology analyzer (Sysmex Corporation, Hyogo, Japan), and procalcitonin, CRP, and albumin parameters on an AU 5800 chemistry analyzer (Beckman Coulter Inc. Brea, California, USA). Presepsin levels were measured on an Architect I2000SR immunoassay analyzer (Abbott Laboratories, Illinois, USA) using a human presepsin (PSPN) Elisa kit (Shanghai YL Biotech Co. Ltd.).

### Statistical analysis

The Statistical Package for the Social Sciences (SPSS) for Windows 22.0 program was used for statistical analysis of variables. Continuous variables were defined as mean±standard deviation and compared by independent samples t-test. Receiver operating characteristic (ROC) curve analysis was performed with presepsin levels.

## RESULTS

The study included 87 patients, aged 22–97 years, who were admitted to the ICU with a diagnosis of sepsis. The mean age of these patients was 72.33±12.51 years. Furthermore, 42 (48.28%) of these patients were female and 45 (51.72%) were male. The pathogens causing sepsis in the patients were as follows: 44 (50.57%) gram-positive bacteria, 34 (39.08%) gram-negative bacteria, and 9 (10.34%) fungi.

The mean length of stay in the ICU of the patients included in the study was 26.07±16.99 days. While 30 (34.48%) of the patients survived, 57 (65.52%) did not. Laboratory findings of survivors and non-survivors are shown in Table 1.

**Table 1:** Laboratory findings of survivors and non-survivors.

Laboratory findings	Survivors	Non-survivors	p
White blood cells (*10^9^/L)	12.92±6.21	14.07±9.74	>0.05
Neutrophils (*10^9^/L)	9.74±5.44	12.86±12.13	>0.05
Lymphocytes (*10^9^/L)	1.16±0.64	1.21±0.75	>0.05
Procalcitonin (μg/L)	5.82±16.99	2.89±9.42	>0.05
C-reactive protein (mg/L)	143.84±90.61	128.48±78.45	>0.05
Albumin (g/L)	23.54±4.99	23.27±5.80	>0.05
Lactate (blood gas) (mmol/L)	2.13±2.30	2.50±2.97	>0.05
Ph	7.36±0.09	7.33±0.11	>0.05
Oxygen saturation (%)	65.28±12.99	70.22±11.69	>0.05
Partial arterial oxygen pressure (mmHg)	43.06±22.02	46.11±22.14	>0.05
Base deficit (mmol/L)	-1.34±6.86	-0.97±7.96	>0.05
Presepsin (pg/mL)	421.50±255.16	823.20±470.86	<0.001

Presepsin levels were higher in non-survivors than in survivors (Table 1). There was no significant difference in other laboratory findings (Table 1).

Binary logistic regression analysis was performed to determine the predictive value of presepsin levels for mortality. As a result of the analysis, the predictive value of presepsin levels for mortality was found to be 78.20%. In addition, the odds ratio was 81.836 (95%CI 8.436–793.867) (p<0.001).

ROC curve analysis was performed to determine the sensitivity, specificity, and cutoff value of presepsin levels in predicting mortality. The area under the curve (AUC) value was 0.819, the sensitivity rate was 73.70%, the specificity rate was 73.30%, and the cutoff value was 612.70 pg/mL. In addition, the positive predictive value was 0.5735 and the negative predictive value was 0.8512.

## DISCUSSION

In recent years, many studies have been conducted to discover biomarkers that can be used in the diagnosis, treatment, and prognostic evaluation of sepsis, and more than 250 biomarkers have been identified to date. Although the heterogeneity of the sepsis process and the increased sensitivity of various detection techniques have led to the emergence of new biomarkers, specific diagnostic biomarkers and effective treatment approaches for sepsis are still lacking in clinical practice^11^. Despite a better understanding of the pathophysiology of sepsis, a translational gap remains to improve the clinical diagnosis of sepsis. Most of the biomarkers proposed for the diagnosis of sepsis do not have sufficient specificity and sensitivity to be used in routine clinical practice. There has been no progress in diagnostic tools due to the focus on the inflammatory pathway. Inflammation and coagulation are known to be associated with the innate immune response^12^. Sepsis is a disease consisting of two concurrent phases, usually with an initial phase of immune activation followed by a chronic immunosuppressive phase leading to immune cell death. The lack of specific treatments for sepsis, a leading cause of morbidity and mortality, is often due to limited knowledge of the immune physiology associated with the disease^13^.

A review of 258 sepsis biomarkers reported that most biomarkers were evaluated together or compared with CRP and/or procalcitonin. In addition, biomarkers with AUC values >0.80 were reported to be potentially more interesting for further studies^14^. Sepsis treatment should be personalized and based on an approach determined using biomarkers to tailor treatment to the needs of each patient. The key characteristics of such biomarkers should be high specificity, sensitivity, and the ability to monitor the progression of sepsis. Since procalcitonin, CRP, and interleukin-6 are secreted in noninfectious processes, their use for this purpose is limited^15^. A recent study found the AUC value of procalcitonin in predicting mortality in patients with sepsis to be 0.608, and it was reported that procalcitonin levels are important in distinguishing cases of gram-negative bacteremia from other causative pathogens^16^. In a multicenter international European prospective study, it was reported that the AUC value for procalcitonin was less than 0.80, although the majority of sepsis-2 cases were caused by gram-negative bacteria^17^. In this study, the AUC value of presepsin in predicting mortality in patients with sepsis was found to be 0.819, and it was reported that there was no statistical difference between the procalcitonin and CRP levels of survivors and non-survivors.

In a study in which sepsis was diagnosed according to the Sepsis Guideline criteria and no pathogenic factors were specified, it was reported that CRP, procalcitonin, and presepsin were not sufficient to predict mortality in the ICU^18^. On the other hand, the sensitivity and specificity of various screening tools used to define sepsis, such as the SIRS criteria and the qSOFA score, are not perfect and should be used with caution^5^. Measurements of procalcitonin and CRP alone at a single time point in ICU patients are not useful in excluding bacterial coinfections^19^. In contrast, numerous multicenter and prospective studies have reported that presepsin levels are higher in patients with bacterial infections. In addition, cutoff values between 600 and 864 pg/mL have been reported to have a sensitivity of 70–87% and a specificity of 63–81%^20^. In this study, which evaluated 89.66% of bacterial and 10.34% of fungal sepsis cases, the sensitivity of presepsin levels in predicting mortality was 73.70%, the specificity was 73.30%, and the cutoff value was 612.70 pg/mL.

Presepsin plays an important role in the development and response of the immune system to infection and as an early marker of sepsis in both adult and pediatric patients^21^. Various immunological biomarkers have been evaluated to develop the best indicator of infection, and presepsin is a new, emerging early indicator for the detection of various infections. In addition, it is a new, non-culture-based technique that improves the identification of infections^20^. Plasma presepsin levels are associated with abnormal CD14 and human leukocyte antigen-DR expression on monocytes. Monitoring presepsin levels may be useful in assessing impaired innate immune response^22^. Upon stimulation with the bacterial agonist lipopolysaccharide, presepsin levels increase early in peripheral mononuclear cells and in a human cell line composed of monocytic cells. This may confirm the potential usefulness of presepsin as an early marker of infectious disease^23^. In addition, presepsin levels increase in patients with fungal sepsis and show a positive correlation with disease severity. Presepsin may be a useful biomarker for sepsis secondary to fungal infections^24^.

## CONCLUSION

In this study, the cutoff value of presepsin levels for mortality in sepsis patients was determined to be 612.70 pg/mL and the AUC value was 0.819. Using this cutoff value, the positive predictive value of presepsin levels for mortality was 0.5735 and the negative predictive value was 0.8512. Therefore, presepsin levels can predict mortality in sepsis.
